# Bacterial survival on inanimate surfaces: a field study

**DOI:** 10.1186/s13104-021-05492-0

**Published:** 2021-03-15

**Authors:** Ruth Hanna Katzenberger, Anja Rösel, Ralf-Peter Vonberg

**Affiliations:** Institute for Medical Microbiology and Hospital Epidemiology, Hannover Medical School, Carl-Neuberg-Str. 1, 30625 Hannover, Germany

**Keywords:** Nosocomial transmission, Bacterial survival, Environment, Inanimate surface

## Abstract

**Objective:**

Environmental surfaces may serve as potential reservoirs for nosocomial pathogens and facilitate transmissions via contact depending on its tenacity. This study provides data on survival kinetics of the most important nosocomial bacteria on a panel of commonly used surfaces. Type strains of *S. aureus*, *K. pneumoniae*, *P. aeruginosa*, *A. baumannii*, *S. marcescens*, *E. faecium*, *E. coli*, and *E. cloacae* were suspended in 0.9% NaCl solution at a McFarland of 1 and got then plated via cotton swabs either on glass, polyvinyl chloride, stainless steel, or aluminum. Surfaces were stored at regular ambient temperature and humidity to simulate routine daycare conditions. Sampling was performed by contact plates for a time period of four weeks.

**Results:**

The longest survival was observed for *A. baumannii* and *E. faecium* on all materials (at least four weeks). *S. aureus* remained viable for at least one week. Gram negative species other than *A. baumannii* were usually inactivated in less than two days. Nosocomial transmission of the above mentioned bacteria may easily occur if no appropriate infection control measures are applied on a regular daily basis. This might be of particular importance when dealing with outbreaks of *A. baumannii* and *E. faecium*.

## Introduction

Frequently touched environmental surfaces are described as a major factor of nosocomial transmission [1, 2] and the probability of nosocomial spread in those events may be influenced by the tenacity of the particular type of microorganism. Bacteria may highly differ in their potential to survive on such surfaces, but up to now there are only few data available on this topic.

There are some reports on estimations of survival times, but those vary extensively with respect to the inoculum, ambient conditions, and the mode of sampling [3]. So for a better understanding of the true risk of nosocomial transmission, there is a need to better characterize bacteria with respect to environmental survival in a more standardized matter.

The Worldwide Outbreak Database [4] is the largest collection of nosocomial outbreaks and contains currently (August 2020) 3,632 nosocomial outbreak reports. According to this database, the following bacteria play the major roles in outbreak events: *S. aureus* (431 outbreaks; 11.9%), *K. pneumoniae* (288; 7.9%), *P. aeruginosa* (259; 7.1%), *A. baumannii* (253, 7.0%), *S. marcescens* (168, 4.6%), *E. faecium* (131, 3.6%), *E. coli* (86; 2.4%), and *E. cloacae* (82; 2.3%).

This study was carried out to determine the capability of those most relevant nosocomial bacteria to persist over a prolonged period of time on various surface materials.

## Main text

### Bacteria

Test organisms were obtained either form the American Type Culture Collection (ATCC) or from the Deutsche Sammlung von Mikroorganismen (German Collection of Microorganisms; DSM). The following type strains were used in the study at hand: *S. aureus* ATCC25932, *K. pneumoniae* ATCC700603, *P. aeruginosa* ATCC27853, *A. baumannii* DSM30011, *S. marcescens* DSM12485, *E. faecium* ATCC19434, *E. coli* ATCC25922, and *E. cloacae* ATCC13047.

Bacterial suspensions were prepared for each of those eight test organisms from fresh overnight cultures at 37 °C under standard conditions on Columbia 5% sheep blood agar (Becton Dickinson GmbH, Heidelberg, Germany). Colonies from the agar were transferred to the liquid suspension until a McFarland turbidity of 1.0 was reached. Bacteria were suspended in 0.9% NaCl solution in order to avoid potential toxic components that may lead to an accidental primary inactivation. In pre-experiments this amount of microorganisms proved sufficient for growing as a bacterial lawn on contact plates used immediately after plating the suspension.

### Surfaces

Survival of the bacteria was tested on glass, polyvinyl chloride (PVC), stainless steel, and aluminum as these materials are frequently used as surfaces in the hospital setting. PVC and other plastic materials are commonly found in form of light switches, shelf spaces for patients, cupboards in bathrooms, bed rails and alarm buttons at the patient´s site. Aluminum may be use for manufacturing hand rails or buttons of elevators. Stainless steel surfaces are very common in doorknobs and levers or in surfaces for the preparation of intravenous infusions or disposal of excretions. Glass surfaces are found on tablet PCs, mobile phones and other touch screens.

**Fig. 1:**
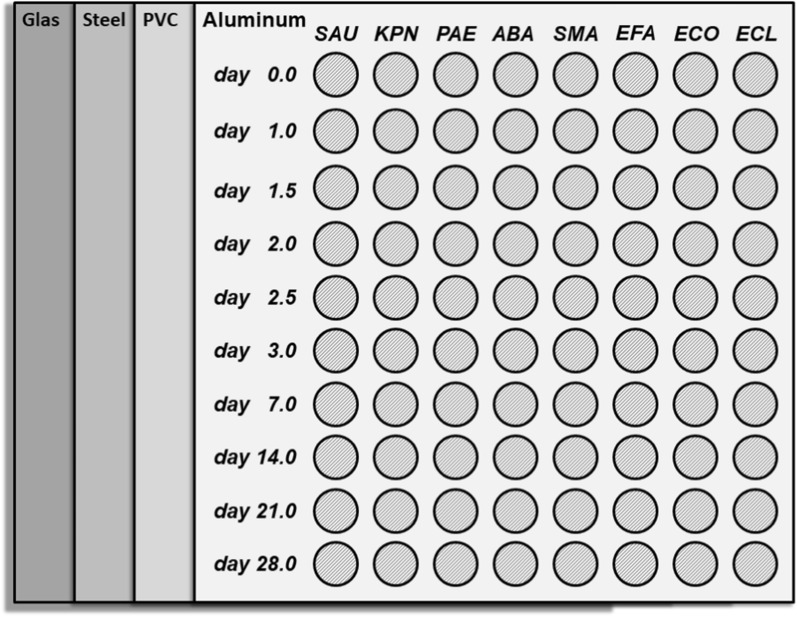
Arrangement of the sampling spots on the various test surfaces. Every bacterial species was sampled at ten different time point on each type of surface (SAU = *S. aureus*; KPN = *K. pneumoniae*; PAE = *P. aeruginosa*; ABA = *A. baumannii*; SMA = *S. marcescens*; EFA = *E. faecium*; ECO = *E. coli*; ECL = *E. cloacae*)

Surfaces were thoroughly decontaminated using 70 Vol-% ethanol directly prior usage. For artificial surface contamination, a volume of 25 µL of the bacterial suspensions circulated by pre-soaked cotton swabs was used per spot to ensure that the entire volume remained on the surface. Ten spots per species and surface were prepared for multiple sampling options at different time points (Fig. 1). Surfaces were stored uncovered on the top of wall cupboards at room temperature (21 °C) at a relative humidity of 31 to 35% in order to maintain conditions as given in the routine daycare of patients on a hospital ward.

### Sampling

Replicate Organism Dectection And Counting (RODAC; Oxoid Deutschland GmbH, Wesel, Germany) contact plates with a contact surface of 25 cm^2^ each were used for sampling over a total period of four weeks. Sampling was primary performed immediately after plating and complete drying of the suspension (day 0) and thereafter on day 1, day 1.5, day 2, day 2.5, day 3, day 7, day 14, day 21, and day 28. Contact plates were then incubated overnight at 37 °C.

### Evaluation

The number of recovered colony-forming units (CFU) was determined visually on each plate. If necessary, subcultures of colonies were prepared on an additional Columbia 5% sheep blood agar in order to differentiate between relevant species and environmental contaminants. The experiment was independently carried out thrice (overall 960 samples) and the mean number of CFU from each sampling spot was calculated. For a conservative calculation of the survival time, a value of only 250 was used for further calculation whenever observing a bacterial lawn (uncountable number of CFU).

## Results

**Fig. 2:**
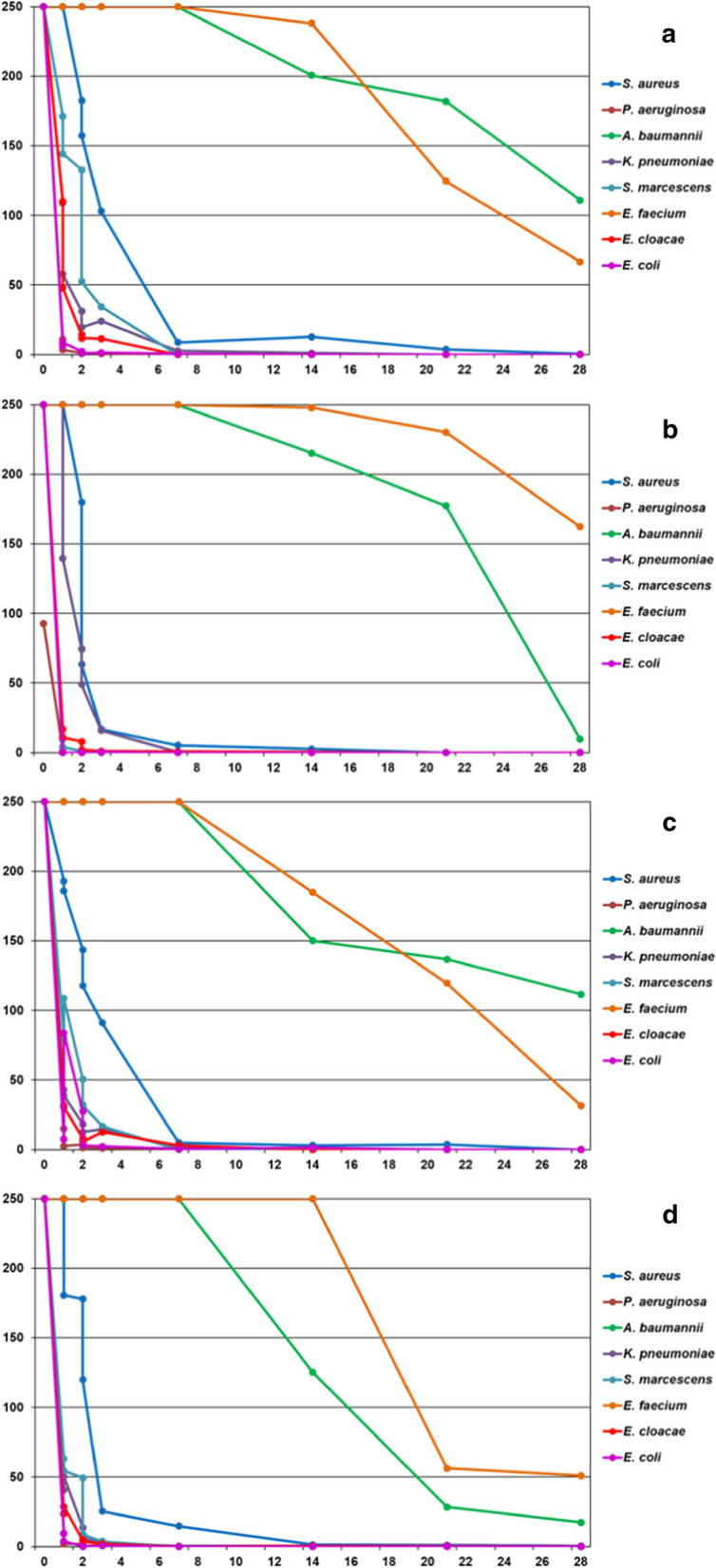
Survival of different bacterial species on **a** glass, **b** stainless steel, **c** polyvinyl chloride, and **d** aluminum

Figure 2 shows the survival kinetics of the test organisms on the four different types of surfaces. Note that *A. baumannii* and *E. faecium* showed the highest survival capability regardless of the material of the surface. Viable bacteria of those two species remained detectable even at the end of the entire observation time period of one month. In contrast, survival of all other species was limited to a few days only.

However, there were also differences within this rather short surviving panel of species. Gram negative bacteria other than *A. baumannii* presented with shortest survival times, e.g. *P. aeruginosa* was completely inactivated in less than two days, while *S. aureus* remained viable for at least a week on all surface materials tested.

## Discussion

Obviously, the length of bacterial survival in the environment impacts the risk of spread. The corresponding time frame depends on multiple factors among them the bacterial species [5] and overall bioburden [6, 7], the source of isolation [5], the type of surface material [8, 9], the ambient temperature [8, 10–13], the extent of UV radiation [14], the local pH [13], the relative air humidity [8, 11], the availability of water and nutrients [8], the presence of chemical noxa [15], the company by additional (concurrent) bacterial species [11] and other factors like pigmentation [16], and biofilm formation [17].

Table 1 provides a summary of studies on survival times of bacteria in vitro under various conditions. However, most of the results from such previous experiments rely on a rather artificial environment setting, while the study at hand determined the tenacity of nosocomially highly relevant species under conditions as existent in routine daycare of patients. Doing so, we could show that especially *A. baumannii* and *E. faecium* are prone for environmental spread in the hospital. This is of importance as antibiotic resistant strains of those two particular species were recently classified as high priority (*E. faecium*) of even critical priority (*A. baumannii*) for health-care settings by the WHO [18]. Long-term transmission via environmental contamination in the endemic setting and several outbreaks caused by *A. baumannii* [19–22] and *E. faecium* [23–25] are extensively described in the medical literature. Furthermore, D'Sousa et al. identified that *A. baumannii* and *E. faecium* even establish synergistic biofilms in vitro when co-cultured [26], which increases the likelihood of prolonged persistence and will facilitate further spread. Thus, our findings confirm the importance of proper infection control measures with emphasis on surface disinfection and/or decontamination procedures.

**Table 1:** Comparison of the findings of other studies on the survival of bacteria on various inanimate surfaces under different environmental conditions

Pathogen	Methods and results					Refs.
	Surface	Inoculum	Environment	Sampling	Survival	
PAE, EFA	Polypropylene, polystyrene, glass and other specific surfaces	n.m	18–21 °C; 40–70% RH	wet and dry swabs, vortexed in NB or BPS or area was cut out and directly vortexed in BPS	PAE: < 2 days EFA: > 11 weeks	[43]
SAU, PAE, KPN, SMA, ECO (clinical isolates)	Aluminum foil (dry), aqua dest., tap water	Aluminum: log 6.4–7.3/cm^2^ Aqua dest.: log 2.8–3.7/mL tap water: log 3.3–3.9/mL	Aluminum: RT; 40–50% RH Aqua dest: RT Tap Water: RT; 30 °C, 40 °C	Aluminum: Foil was put in NB; serial dilution; plateled on agar plates Water samples: directly plated on agar plates	Aluminum: SAU, KPN, SMA, ECO: ≥ 25 days PAE: < 2 days Aqua dest: SAU: < 5 days PAE: < 4 days SMA: ≥ 25 days ECO: < 24 days Tap water: SAU: < 7 days (RT), < 2d (30 °C, 40 °C) PAE: ≥ 12 days ECO: ≥ 12 days (RT), < 5 days (30 °C), < 1d (40 °C) SMA: ≥ 12 days (RT), < 7 days (30 °C), < 2 days (40 °C)	[44]
SAU, PAE, ECO	Dust	10^6^ CFU in NB diluted with aqua dest	0%, 32%, 42%, 58%, 99%	Culture of samples on China blue lactose agar	SAU: 0.6–5.4 m (> 0% RH); > 7.6 m (0% RH) PAE: 5.7–11.9 m (< 99% RH); > 16.9 m (99% RH) ECO: 4.5–11.8 m	[45]
ABA (clinical isolates and type strains)	Glas coverslips	2 × 10^7^ CFU in 20 µL of bovine serum albumin or distilled water	22 °C; 10%, 31%, 93%	Coverslips were vortexted in sterile distilled water	30 days (clinical strain) 2 days (ATCC strain) 60 days (suspended in bovine serum albumin) 11 days (suspended in distilled water) 11 days (31% RH) 4 days (10% RH)	[46]
SAU, PAE, ECO (type strains)	Polymer w/o silver-impregnated	≥ 10^6^–10^7^ CFU dry/liquid inoculum	37 °C; humid chamber	Neutralizing silver by TSB and horse serum, dilution on agar, filtration on cellulose nitrate membrane	SAU: ≤ 7 days (dry inoculum); > 7 days (liquid inoculum) PAE: ≥ 7 days (better survival in liquid inoculum) ECO: ≤ 7 days (data for dry inoculum only available)	[47]
PAE, KPN, SMA, ECO (clinical and environmental strains)	Different textiles such as cotton, polyester and polyethylene	10^2^ CFU 10^4^–10^5^ CFU	22.5–26.2 °C 20–49% RH	Incubation in thioglycolate bouillon	PAE: < 1 h-–7 h (inoculum 10^2^ CFU) 2 h–7 days (inoculum 10^4^–10^5^ CFU) KPN: 1–3 days (inoculum 10^2^ CFU) 4–32 days (inoculum 10^4^–10^5^ CFU) SMA: < 1–2 h (inoculum 10^2^ CFU) 12 h–10 days (inoculum 10^4^–10^5^ CFU) ECO: < 1–8 h (inoculum 10^2^ CFU) 13 h–36 days (inoculum 10^4^–10^5^ CFU)	[6]
SAU (MRSA and MSSA), EFA (VRE and VSE)	Different textiles (cotton, polyester, polyethylene, other)	4.1 × 10^5^ CFU	22.9–24.5 °C; 30–49% RH	Incubation in thioglycolate bouillon	SAU: 1– > 90 days EFA: 22–> 90 days	[7]
EFA (VRE; clinical isolates)	Various environmental surfaces	10^2^ / 10^4^ CFU	n.m	Rodac contact plates	Countertops (10^4^ CFU): 7 days Bedrails (10^4^ CFU): 1 days Telephone (10^2^ CFU): 1 h Stethoscope (10^2^ CFU): 0.5 h	[48]
PAE (clinical, environmental, mucoid and non-mucoid strains)	Sterile petri dish	5 × 10^6^ CFU in saline on 6 cm^2^	n.m	Sampling with moistened sterile cotton swabs, vortexed in NB, serial dilution, cultured on blood agar	≥ 2 days (most mucoid and non-mucoid strains)	[49]
SAU, PAE, KPN, ECO (laboratory strains and wild type)	White laminate surface (soiled, clean)	3 × 10^2^ CFU in water or broth	30° C; 40–45% RH	Tryptone soya agar contact plates	Soiled: SAU ≥ 24 h (laboratory strain and wild type) PAE ≥ 24 h (laboratory strain) KPN < 24 h (wild type) ECO ≤ 24 h (laboratory strain and wild type) Clean: SAU ≤ 24 h (laboratory strain and wild type) PAE ≤ 24 h (laboratory strain) KPN ≥ 24 h (wild type) ECO ≤ 24 h (laboratory strain and wild type)	[50]
SAU (MRSA: clinical, outbreak, sporadic strains)	Bottels w/o dust	10^9^ CFU in sterile PBS	RT; conventional RH; dust protected	Samples vortexted in PBS before incubation on sheep blood agar	> 6 m (w/o dust); longest survival in outbreak strains	[51]
SAU (MSSA and MRSA)	Bottels w/o dust	10^8^ CFU in sterile PBS	22–27 °C; 27–45% RH; dust protected	Samples vortexted in PBS before incubation on sheep blood agar	MSSA: < 28 days (no dust); shorter with dust MRSA: < 175 days (no dust); < 126 days (with dust)	[52]
SAU, PAE (type strains) ABA (clinical isolate)	Enamel, formica, stainless steel	2.5 × 10^5^ on 8 cm^2^	20–22 °C 60–70% RH	CLED agar contact plates Enamel: swab moistened in sterile saline inoculated onto CLED agar	SAU: 3–10 days PAE: 1–5 days ABA: 6–12 days	[53]
ABA (clinical isolates and type strains)	Ceramic, PVC, rubber, stainless steel	8 × 10^6^ CFU	22 °C; 50% RH; darkness; dust protected	Samples shaked in 0.9% NaCl, membrane filtration and serial dilution	≥ 104 days (isolates from dry sources better than wet sources)	[5]
EFA (VSE and VRE; clinical and environmental isolates)	PVC	10^7^ CFU	22 °C; 50% RH; dust protected	Samples shaked in 0.9% NaCl, membrane filtration and or serial dilution	7 days—> 4 m	[54]
ECO	Glas	“one McFarland suspension” 1:1 diluted in water, saline, sheep blood	RT	Samples vortexed in BHI	≤ 70 days	[55]
ECO (type strain)	Stainless steel, copper, copper-containing alloys	10^7^ CFU	4 °C and 20 °C	Samples vortexed in PBS, serial dilution, pipetted onto nutrient agar	> 28 days (stainless steel; for both temperatures) 1.5 h (copper at 22 °C); 4.5 h (copper at 4 °C) < 2 h (copper nickel alloy at 20 °C); < 6 h (copper nickel alloy at 4 °C)	[56]

In recent years there were innovative attempts to reduce the bacterial burden on frequently touched surfaces in hospitals, for example by coating them with layers containing direct bactericide substances or chemicals that diminish biofilm formation [27–29]. Another rather novel sanitation strategy is the use of (non-pathogenic) probiotic bacteria that are capable of reducing in a stable way the surface load of pathogens [30] or the use of UV-C light for surface decontamination [14]. However, all of those approaches are still far from comprehensive use in hospitals worldwide so the significance of traditional cleaning and surface disinfection measures will most likely continue for decades.

## Conclusion

Nosocomial transmission of *A. baumannii* and *E. faecium* via contaminated surfaces may easily continue for several weeks if no appropriate infection control measures are applied. However, we could show that all nosocomially relevant pathogens may survive for a few days and thus represent a relevant risk for transmission within the hospital. So, in an outbreak infection control personnel should thoroughly search for so far unidentified areas or for breaches in standard decontamination procedures if pathogen spread continues despite high efforts in cleaning and disinfection.

## Limitations

### Generalization of results

Obviously, there are some limitations to our study that need to be addressed. First of all we only tested one single strain of each species. Therefore generalization of our findings should be done with caution. However, Jawad et al. compared the survival times for a total of 39 *A. baumannii* isolates (22 strains from nosocomial outbreaks and 17 sporadic strains). Their results in terms of survival time were comparable to our findings, but they failed to observe statistically significant inter-lineage differences with respect to bacterial tenacity (26.5 vs. 27.2 days) [31]. On the other hand, there is some newer data suggesting that hydrophilic clonal lineages of *A. baumannii* possess thicker cell walls and, thus exhibited higher resistance to desiccation compared to hydrophobic strains. This could provide an advantage in environmental survival [32]. Drying resistance of *A. baumannii* may also depend on mutations and expression of the two-component response regulator gene bfmR, which is important for its virulence and also for the expression of stress-related proteins during a stationary phase [33]. This topic needs to be examined for *A. baumannii* and the other species alike in more detail in future studies.

### Biofilm formation

Secondly, we did not check for the degree of biofilm formation although this may also influence the ability to survive on an inanimate surface [34]. For example, *A. baumannii* may form strong biofilms on stainless steel surfaces and bacteria within this biofilm are significantly more resistant to environmental noxa than are their planktonic counterparts [35]. *E. faecium* may also develop biofilms regardless of a concomitant drug resistance but more often in the presence of the esp gene [36–39]. Ghaziasgar et al. observed this ability even significantly more often in nosocomial isolates while it was less common in wild type strains outside the hospital (100% vs 75.6%; p < 0.05) [40].

### Adaptation and virulence of pathogens

Finally, we only measured the number of recovered bacteria via contact plates. Thus, we do not know whether or not changes in the virulence of a pathogen occurred. Although such a phenomenon would not directly affect the transmissibility, it would still be of clinical relevance. Chapartegui-Gonzalez et al. tested five clinical isolates of *A. baumannii* in long-time survival experiments under simulated hospital conditions. All strains were able to rapidly adapt to both the temperature shift and nutrients availability and maintained their virulence factors despite starvation and desiccation [41]. Once again, similar circumstances apply for enterococci, too [42]. We therefore assume that there was no significant reduction of virulence in the strains used in our study.

### Reduction of bioburden by regular decontamination of surfaces

If performed properly, a thorough cleaning and disinfection will significantly reduce the risk of pathogen spread regardless of its tenacity. Unfortunately, breaks in the correct cleaning process are commonly observed due to various reasons. Furthermore small damages to surfaces may cause tiny notches that are then difficult to decontaminate. That is why there are several outbreaks caused by insufficient surface decontamination available in the medical literature. Therefore, this study once again stresses the importance of thorough and regular decontamination of frequently touches surfaces in the hospital for the sake of the safety of patients.

## Data Availability

All data generated or analysed during this study are included in this published article and its supplementary information files.

## Abbreviations

ABA: *Acinetobacter baumannii*
BHI: Brain heart infusion
BPS: Buffered peptone saline
CFU: Colony forming units
CLED: Cysteine lactose electrolyte deficient
d: Day
ECO: *Escherichia coli*
ECL: *Enterobacter cloacae*
EFA: *Enterococcus faecium*
h: Hour
KPN: *Klebsiella pneumoniae*
m: Month
MRSA: Methicillin resistant *Staphylococcus aureus*
MSSA: Methicillin susceptible *Staphylococcus aureus*
NB: Nutrition broth
n.m.: Not mentioned
PAE: *Pseudomonas aeruginosa*
PBS: Phosphate-buffered saline
PVC: Polyvinyl chloride
RH: Relative humidity
RT: Room temperature
SAU: *Staphylococcus aureus*
SMA: Serratia marcescens
TSB: Trypticase soy broth
VRE: Vancomycin resistant enterococcus
VSE: Vancomycin susceptible enterococcus
w/o: With and without
